# Supplementary material for: Mechanistic and structural basis for activation of cardiac myosin force production by omecamtiv mecarbil
Source: Nat Commun. 2017 Aug 4;8:190. doi: 10.1038/s41467-017-00176-5 (PMC5543065; doi:10.1038/s41467-017-00176-5)
Supplement: Supplementary file 1 — Supplementary Information [file 41467_2017_176_MOESM1_ESM.pdf]

File name: Supplementary Information
Description: Supplementary figures, supplementary tables, supplementary note and supplementary references.

File name: Supplementary Movie 1
Description: Comparison between the PR and PPS OM binding sites. Superimposition of the cardiac PR and PPS structures containing OM. The ‘PR’ allosteric binding pocket (orange) is quite distant from the ‘PPS’ binding site (bright pink) for OM. Both structures are compared using the N-ter subdomain (dark grey) as a reference.

File name: Peer review file
Description:

Supplementary Note 1

The loops and linkers of the transducer are compliant and can be influenced by crystal packing

The transducer corresponds to structural elements at the center of the motor domain, in particular the central beta-sheet, that undergo important conformational changes along the transitions that produce force[1–4] (Fig. 1b). It has been proposed by Winkelmann et al[5] that OM binding could allosterically influence the transducer conformation and thus modulate force production. However, our analysis of this region in different cardiac crystal structures shows that this hypothesis is unlikely. Indeed, there is no conformational difference in the residues that connect the drug binding site (either the 'PR' or the 'PPS' sites) and this transducer region. In fact the twist of the central beta-sheet itself is unchanged whether OM is bound or not in the PR state (Supplementary Fig. 5a). Only distal rearrangements are observed in transducer loops and linkers when PR cardiac structures are compared[5] but they occur independently of drug binding and reflect rather local influence on the conformation of these regions which are involved in contacts with other molecules in the crystal. The study of the inter-molecular interactions of the transducer residues show that the differences observed among the two molecules of the asymmetric unit in the PR state (4PA0) are well accounted for by the contacts these residues make in the crystal packing (Supplementary Fig. 5b). They are not linked to differences in the occupancy of the drug binding site. This is corroborated by the fact that the transducer in molecule B (4PA0)[5] in which OM is bound adopts a similar conformation than those found for the apo structure (4P7H)[5]. Moreover, in the states myosin populates at the beginning of the powerstroke, the OM-S1-PPS and *Argopecten* striated muscle (scallop) myosin II PPS (1QVI[6]) structures indicate that the transducer is not much affected by drug binding. In addition to these structural arguments, a recent CryoEM structure[4] revealed that the ADP release step corresponds to major changes in the transducer. However, ADP release is unaffected by OM which does not support a direct influence of OM binding on the transducer conformation. Moreover, the P_i release transition occurs without much change in the transducer[7] while OM clearly accelerates the rate of this step. These results indicate that OM is not likely to modulate force production via a direct control on the transducer structural rearrangements during force generation.

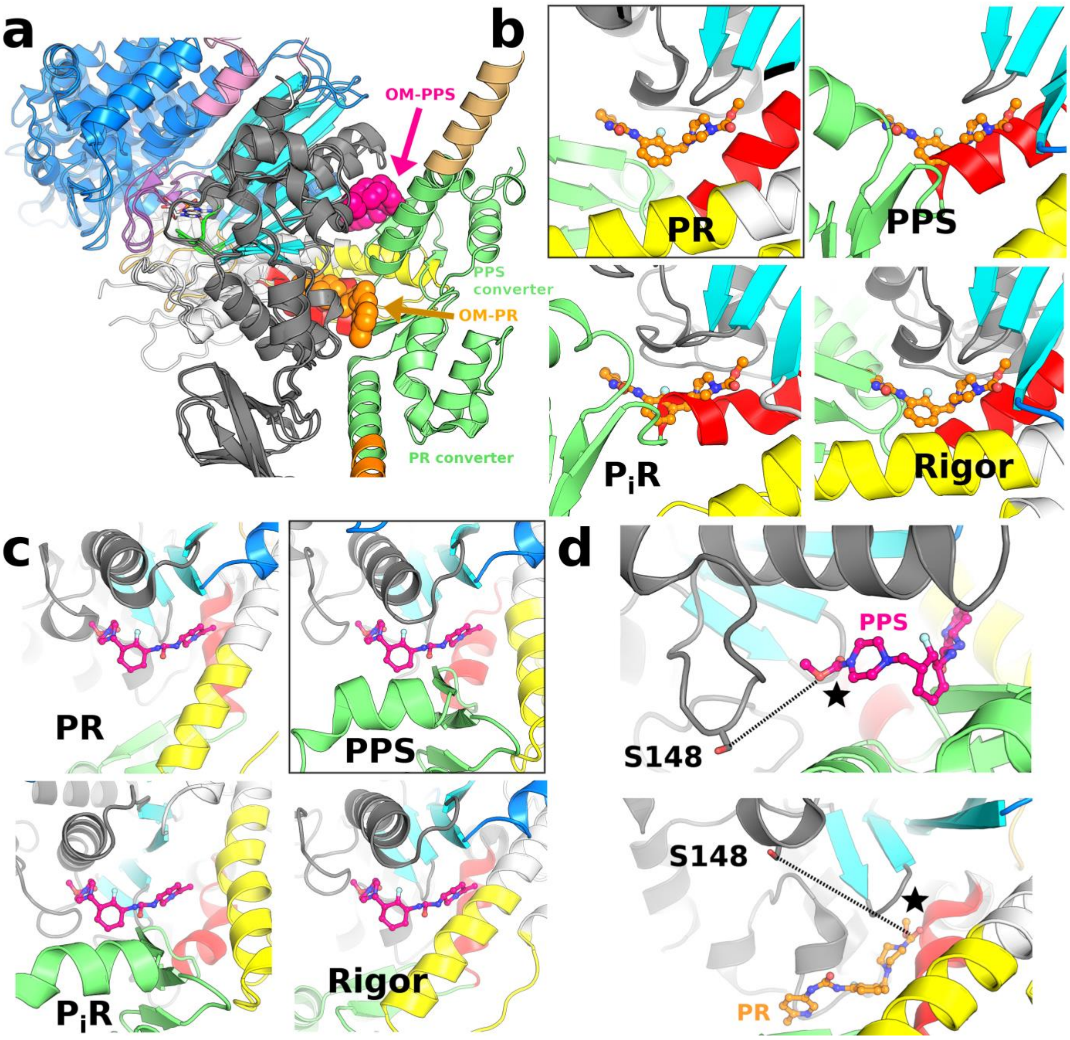

Supplementary Figure 1 – Comparison of the OM binding pockets

(a) The 'PR' allosteric binding pocket for OM (orange) and the 'PPS' binding site for OM (bright pink) are quite distant from one another. Superimposition of the cardiac PR (4PA0) and PPS structures using the N-terminal subdomain as a reference (dark grey). See also Supplementary movie 1. **(b)** Detail of the 'PR' binding pocket. OM is found near the SH1 helix (red) and the converter (green). The structural elements surrounding the 'PR' OM binding pocket as found in the OM-MD-PR structure (4PA0)[5] (black contour) are shown for four states of the myosin motor: **PPS** (pre-powerstroke, **OM-S1-PPS** structure), **P_iR** (myosin VI P_i release, 4PFO[7]) and **Rigor** (myosin IIb, 4PD3[8]). Note that the 'PR' OM pocket is closed in all states except in the PR state. All the structures were aligned using the N-terminal subdomain as a reference. **(c)** Detail of the 'PPS' pocket as found in the OM-S1-PPS structure (black contour) and using the same structures as in (b). In this case, the 'PPS' allosteric site is formed in the PPS and the P_iR states for which the converter (green) is in position to make specific contacts with OM. In the Rigor and PR states, the relay and converter are found in an unprimed position and cannot participate in closing the OM 'PPS' pocket, in agreement with the fact that OM cannot bind strongly in these states. **(d)** The benzophenone derivative of OM shown to react with Ser148[9] has the reactive group attached on the carboxymethyl-piperazine moiety of OM (black star). Interestingly, this group is found near Ser148 in the PPS state (8.4 Å) but it is too far (16 Å) from Ser148 in the 'PR' allosteric pocket to account for the observed reactivity. The dotted lines represent the distance between the serine and the part of the OM molecule to which the benzophenone is attached.

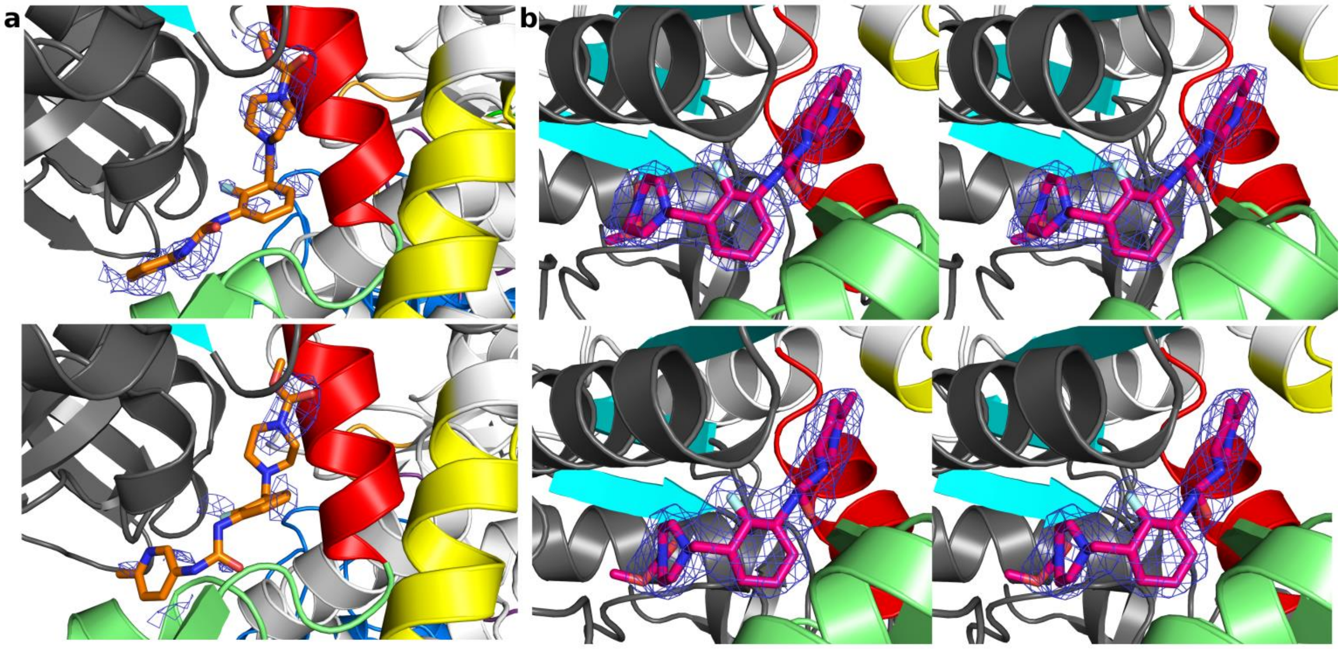

Supplementary Figure 2 - Composite 2mFo-DFc omit maps at 1σ showing the quality of the OM densities. **(a)** OM density near the OM 'PR' binding site calculated from the 4PA0 structure which contains two molecules per asymmetric unit (upper panel, molecule A; lower panel, molecule B). The electron density for OM bound to the cardiac myosin motor domain in the PR state is fragmented likely due to the partial occupation of the site. **(b)** Stereo-image of the OM density bound to the motor domain in the PPS state from the OM-S1-PPS structure that contains two molecules per asymmetric unit (upper panel, molecule A; lower panel, molecule B). In order to minimize the phase bias, the composite omit maps were calculated with the annealing method, using the same parameters in both cases.

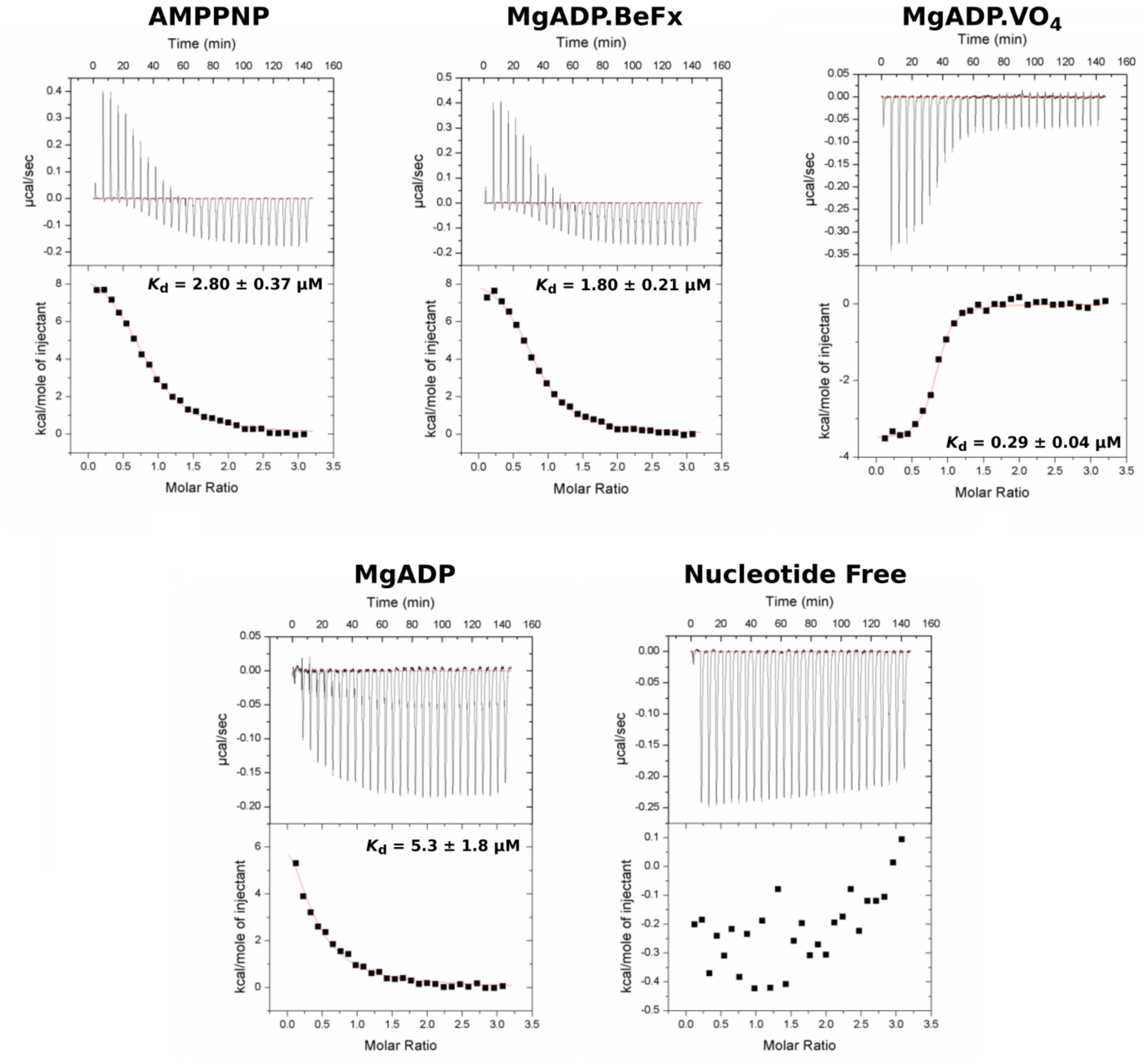

Supplementary Figure 3: ITC binding experiments.

The raw data for ITC experiments presented in Table 2 is shown here. The affinity for OM binding to cardiac myosin is evaluated depending on the nucleotide-bound to the motor domain.

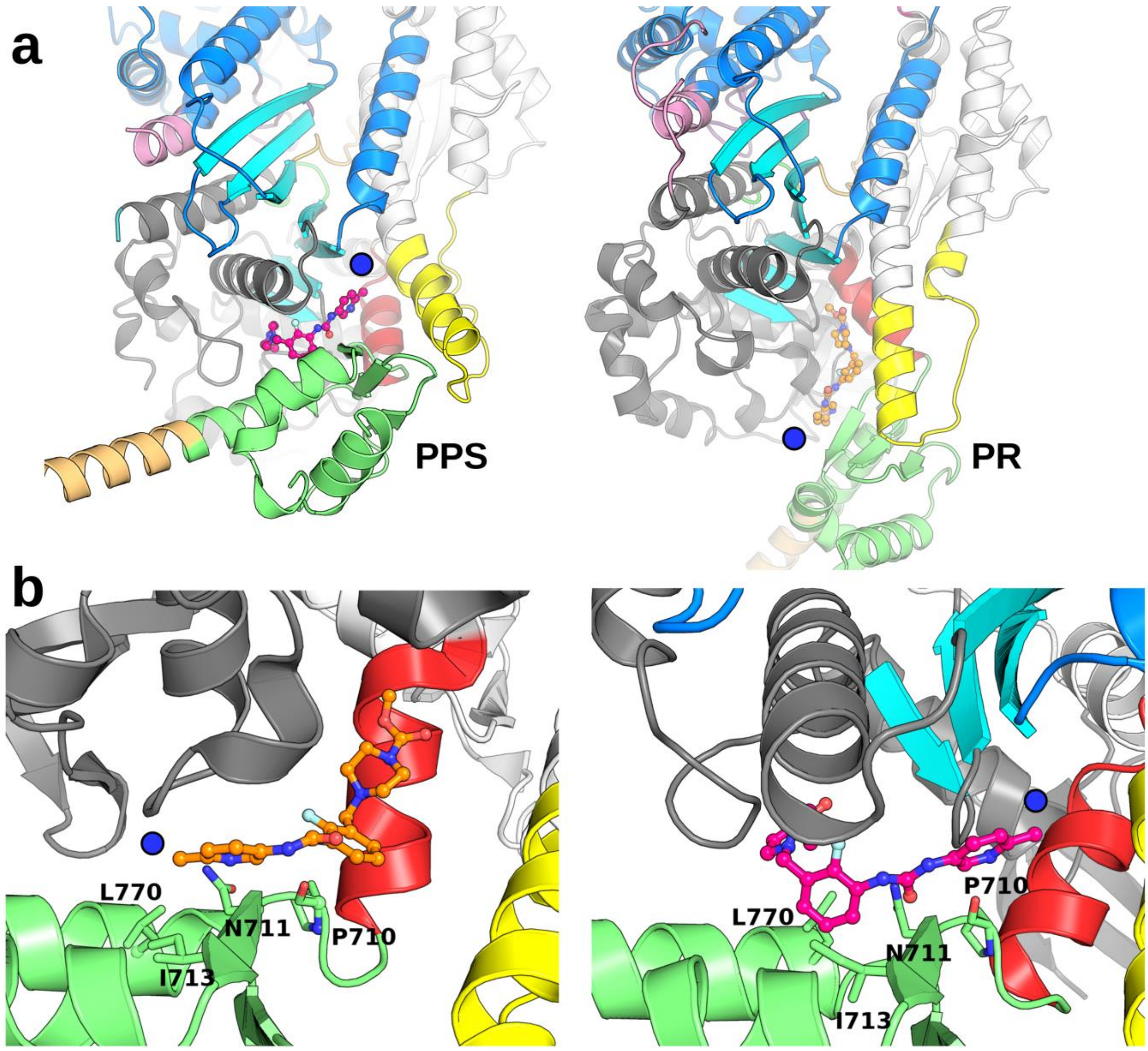

Supplementary Figure 4 – Orientation of the OM molecule in the 'PR' and the 'PPS' binding sites.

(a) In the PPS state (left), the converter (green) traps and stabilizes OM binding. In the PR state (right), the drug binds in a different pocket and makes limited contacts with the converter. Note in addition that the OM molecules have an opposite polarity when bound in these two sites (the blue dot highlights the position of the methyl-pyridinyl ring group of the OM molecule). **(b)** Only four common residues are found interacting with OM in both structures. Detailed analysis of the pocket reveals that these common residues (P710, N711, I713 and L770) belong to the converter and interact with different parts of the OM molecule in these two structures: **(1)** the carbonyl oxygen of Pro710 interacts with the carbamoyl-amino linker in the PR, and with the methyl-pyridinyl ring in PPS; **(2)** Asn711 interacts with the methyl-pyridinyl N1 in PR, while in PPS it makes an hydrogen bond with O1 in the carbamoyl central linker; **(3)** Ile714 and **(4)** Leu770 both interact with the methyl-pyridinyl ring in the PR structure, while they interact with the carboxymethyl-piperazine and the fluoro-benzyl ring in PPS, respectively.

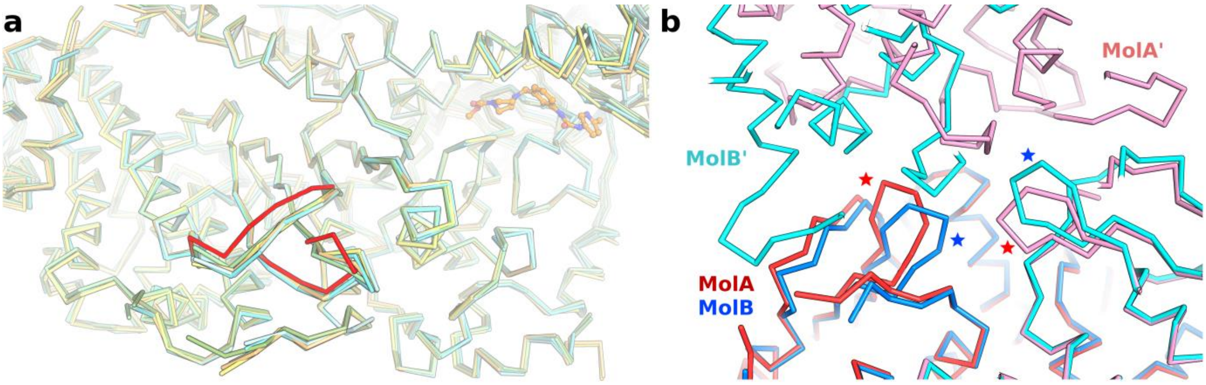

Supplementary Figure 5 – OM does not allosterically influence the transducer conformation

(a) 4PA0 (PR-OM), 4P7H (PR-APO without nucleotide) and 4DB1 (PR-APO with Mn-AMPPNP) molecules are superimposed using the N-terminal and the U50 subdomains as a reference. There are no differences in all the molecules from the OM pocket until the transducer loops (shown in red for 4PA0 molA), indicating that drug binding doesn't influence the conformation of these distal loops.

(b) In 4PA0, both molecules (molA and molB) have different environments surrounding the transducer loops (red and blue stars) linked to the crystal packing. These differences account for the loop conformations observed in the crystal. MolA' and MolB' are the crystal mates of MolA and MolB, respectively. It is clear that MolB' is not compatible with the conformations of the loop found in MolA. This study of the packing thus supports the statement that these loops are easily influenced by inter-molecular interactions that occur in the crystal.

Supplementary Tables

Supplementary Table 1 – Comparison of the reported Omecamtiv Mecarbil binding sites

	'PR' binding site (4PA0)	'PPS' binding site (5N69)
**Global contact area**	422-444 Å^2 (MolB and MolA)	470-483 Å^2 (MolB and MolA)
**Drug conformation**	elongated with two small bends<br>variable in the two molecules 4PA0	sharp 90° bend<br>both molecules
**Properties of the pocket**	surface pocket of the PR structure<br>hydrophobic + 2 H-bonds<br><br>Drug is buried in a hydrophobic site	formed and stabilized by induced fit, which primes the lever arm position<br>hydrophobic + 5 H-bond<br>Drug is buried in a hydrophobic cavity
**Residues involved in binding**	N-ter (A91, M92,L96, S118, G119, F121)<br>SH1 helix (V698, I702, C705)<br>converter (P710, N711, R712, L770)	N-ter (K146, R147, N160, Q163, Y164, T167, D168)<br>relay helix (H492)<br>converter (P710, N711, R712, I713, R721, L770, Y722, E774)
**Specificity of the pocket**	not specific – 3 homologous changes	highly specific – not conserved among Myo2s
**Electron density map**	missing density for some drug atoms<br>possibly lower occupation of the drug	very good density for all drug atoms<br>Full occupation
**LLDF(*)**	10.75 (MolA), 7.52 (MolB)	-0.54 (MolA), -0.66 (MolB)
**Photo-crosslinking results**	Drug too far away and not in the correct orientation to allow crosslinking with Ser148	Drug position and orientation is coherent with reactivity with Ser148
**Influence on the myosin structure**	binds without much change<br>OM has little influence upon binding	binding induces priming of the lever arm upon cleft closure.<br>Not much influence for the motor domain conformation

(*) The Local Ligand Density Fit value (LLDF) represents the quality of the electron density of the ligand with respect to its neighboring residues. A bigger LLDF value represents a worse ligand electron density. It is calculated by comparing the Real-Space R value (RSR) of the ligand to mean and standard deviation of the RSR of neighboring residues within 5 Å. RSR is calculated as following[10,11] (ρcalc and ρobs refer model and experimental electron density respectively):

$$RSR = \frac{\sum|\rho_{obs} - \rho_{calc}|}{\sum|\rho_{obs} + \rho_{calc}|}$$

Supplementary Table 2 - Sequence conservation in the OM 'PR' binding pocket

			N-ter cavity		Relay helix	SH1	Converter
Residue number #	Gene	Uniprot	91 96	118 121	493 497	698 707	708 713
Hs-βCar-Myo2	*MYH7*	P12883	AMLTFL	SGLF	MFVLE	VLEGIRICRK	GFPNRI
Bt-βCar-Myo2	*MYH7*	Q9BE39	......	....	.....	....	......
Hs-αCar-Myo2	*MYH6*	P13533	......	....	.....	....	......
Hs-Sm-Myo2	*MYH11*	P35749	.E..C.	....	..I..	.........Q	......
Gg-Sm-Myo2	*MYH11*	P10587	.E..C.	....	..I..	.........Q	......
Rb-Sk-Myo2	*MYH4*	Q28641	..M.H.	....	.....	....	...S..
Rb-Sk-Myo2	*MYH13*	Q9GJP9	..M.H.	....	.....	....	...S..
Gg-Sk-Myo2	*N116*	P13538	..M.H.	....	.....	....	...S.V
Hs-NMM2a	*MYH9*	P35579	.E..C.	....	..I..	.........Q	V
Hs-NMM2b	*MYH10*	P35580	.E..C.	....	..I..	.........Q	......
Hs-NMM2c	*MYH14*	Q7Z406	.E..C.	....	.....	.........Q	......
Hs-Myo5a	*MYO5A*	Q9Y4I1	TA.SY.	C.IV	V.K..	...T...SAA	...S.W
Hs-Myo6	*MYO6*	Q9UM54	CS.MY.	VANI	ILKE.	MVSVLDLMQG	.Y.S.A
Hs-Myo10	*MYO10*	Q9HD67	AS..E.	I.SI	IFS..	M..TV..RKA	.YAV.R

Sequence comparison of related myosin II family members and some unconventional myosins. The residues directly involved in OM binding in the PR pocket[5] are highlighted in red. Dots (.) represent identical residues. The Hs, Bt, Gg and Rb abbreviations stand for human, bovine, chicken and rabbit myosins, respectively.

Supplementary References

1. Coureux, P.-D. *et al.* A structural state of the myosin V motor without bound nucleotide. *Nature* **425,** 419–23 (2003).

2. Coureux, P.-D., Sweeney, H. L. & Houdusse, A. Three myosin V structures delineate essential features of chemo-mechanical transduction. *EMBO J.* **23,** 4527–4537 (2004).

3. Sweeney, H. L. & Houdusse, A. Structural and functional insights into the Myosin motor mechanism. *Annu. Rev. Biophys.* **39,** 539–57 (2010).

4. Wulf, S. F. *et al.* Force-producing ADP state of myosin bound to actin. *Proc. Natl. Acad. Sci. U. S. A.* **113,** E1844–E1852 (2016).

5. Winkelmann, D. A., Forgacs, E., Miller, M. T. & Stock, A. M. Structural basis for drug-induced allosteric changes to human β-cardiac myosin motor activity. *Nat. Commun.* **6,** (2015).

6. Gourinath, S. *et al.* Crystal Structure of Scallop Myosin S1 in the Pre-Power Stroke State to 2. 6A Resolution: Flexibility and Function in the Head. *Structure* **11,** 1621–1627 (2003).

7. Llinas, P. *et al.* How Actin Initiates the Motor Activity of Myosin. *Dev. Cell* **33,** 401–412 (2015).

8. Münnich, S., Pathan-Chhatbar, S. & Manstein, D. J. Crystal structure of the rigor-like human non-muscle myosin-2 motor domain. *FEBS Lett.* **588,** 4754–4760 (2014).

9. Malik, F. I. *et al.* Cardiac Myosin Activation: A Potential Therapeutic Approach for Systolic Heart Failure. *Science* **331,** 1439–1443 (2011).

10. Jones, T. A., Zou, J. Y., Cowan, S. W. & Kjeldgaard. Improved methods for binding protein models in electron density maps and the location of errors in these models. *Acta. Crystallogr. A.* **47,** 110–119 (1991).

11. Brändén, C.-I. & Jones, T. A. Between objectivity and subjectivity. *Nature* **343,** 687–689 (1990).
